# Protect ya Grandma! The Effects of Students' Epistemic Beliefs and Prosocial Values on COVID-19 Vaccination Intentions

**DOI:** 10.3389/fpsyg.2021.683987

**Published:** 2021-06-24

**Authors:** Tom Rosman, Kathrin Adler, Luisa Barbian, Vanessa Blume, Benno Burczeck, Vivien Cordes, Dilara Derman, Susanne Dertli, Hannah Glas, Virginia Heinen, Stefan Kenst, Marie Khosroschahli, Laura Kittel, Corinna Kraus, Alica Linden, Anastasia Mironova, Lena Olinger, Fatbardh Rastelica, Theresia Sauter, Vera Schnurr, Elisabeth Schwab, Yves Vieyra, Andreas Zidak, Ivana Zidarova

**Affiliations:** ^1^Leibniz Institute for Psychology (ZPID), Research Literacy Unit, Trier, Germany; ^2^University of Trier, Psychology Department, Trier, Germany

**Keywords:** epistemic beliefs, justification by authority, vaccination intentions, prosocial values, COVID-19

## Abstract

The present study investigates epistemic beliefs (beliefs about the nature of knowledge and knowing) and prosocial values as predictors of COVID-19 vaccination intentions. As a first hypothesis, we posit that beliefs in justification by authority will positively relate to vaccination intentions. Second, we expect a positive relationship between prosocial values and vaccination intentions. Third, we hypothesize that beliefs in justification by authority moderate the relationship between prosocial values and vaccination intentions, so that the positive correlation between prosocial values and vaccination intentions becomes stronger with increasing beliefs in justification by authority. Hypotheses were tested in a sample of *N* = 314 German university students, a group with rather high mobility, who, when vaccinated, will increase the chance of attaining herd immunity. Hypotheses were tested using correlational and multiple regression analyses. Results revealed a highly significant positive relationship between justification by authority and vaccination intentions, whereas both hypotheses that included prosocial values did not yield significant results. Additional exploratory analyses revealed that the relationship between justification by authority and vaccination intentions was mediated by beliefs in the safety and effectiveness of the vaccines. Furthermore, significant negative relationships were found between personal justification and vaccination intentions as well as between justification by multiple sources and vaccination intentions. These results highlight the crucial role of science and public health communication in fostering vaccination intentions regarding COVID-19.

## Introduction

Public engagement with science has always been important for individual well-being and for social progress. However, extraordinary times bring with them special circumstances. One such is arguably that of a pandemic sparked by the spread of the SARS-CoV-2 virus. In these times, it becomes particularly obvious how important it is that laypeople engage with scientific knowledge in a nuanced and meaningful way. But how exactly do individuals actually perceive and evaluate scientific knowledge? This question is directed toward epistemic beliefs, defined as individual beliefs about the nature of knowledge and knowing (Hofer and Pintrich, 1997). Over the year 2020, it has become clear what influence individual beliefs about science may have. Scientists and the insights they have gained have moved into the broad focus of the media and thus of the public. Countless discussions have arisen and been fought out. One of them is already in full swing. Now, in spring 2021, no question looms as urgently as that of vaccination intentions: Will enough people have themselves vaccinated in order to curb the spread of COVID-19? In this context, the present study investigates how individual epistemic beliefs, in combination with prosocial values, relate to vaccination intentions regarding COVID-19. All confirmatory hypotheses were developed in a research-oriented psychology course (Master track) at the University of Trier. While we did not formally preregister our study for time reasons, the hypotheses as well as our study design, sampling plan, and analysis plan were specified before collecting the data using a preregistration template.

### The Importance of Vaccinations in the Context of COVID-19

Vaccinations not only protect vulnerable groups from severe COVID-19 (Graeber et al., 2020; Connors et al., 2021; Dagan et al., 2021). In fact, they likely also serve, once that large parts of the population are vaccinated, as a powerful means to curb the spread of the pandemic altogether (Lu et al., 2021). Early evidence suggests that vaccinations reduce the viral load in infected but vaccinated individuals (Levine-Tiefenbrun et al., 2021), and that they may even prevent a large extent of (symptomatic and asymptomatic) infections (Dagan et al., 2021; Hall et al., 2021). In this context, investigating young adults' vaccination intentions seems particularly important. In fact, younger people usually take part in a large range of leisure activities and are in close social contact with a high number of people. Furthermore, recent research by Betsch et al. (2021) suggests that young adults–compared to the elderly–are less likely to reduce their contacts during the pandemic. Hence, although young adults are at a lower risk regarding the viral disease itself (Zhou et al., 2020), them becoming vaccinated is of elementary importance to curb the spread of infections due to their sociability and mobility. Support for this assumption comes from a recent modeling study by Wang et al. (2021), who found that vaccinating the elderly reduces the number of deaths, whereas vaccinating the younger and socially active population minimizes the number of infections. Therefore, once enough vaccine is available to protect at-risk groups, a broad vaccination of younger groups, such as university students, will likely contribute to a better protection of the whole population.

Since COVID-19 vaccinations are voluntary, each and every one's individual willingness to participate in the vaccination campaigns is a key factor in the success of the COVID-19 response (Lu et al., 2021). According to a study by Graeber et al. (2020), the general willingness of the German population to be voluntarily vaccinated against COVID-19 was around 70 percent in June and July 2020. Internationally, similar figures have been found (e.g., Taylor et al., 2020). Furthermore, a serial cross-sectional study by Betsch et al. (2021) recorded the German population's intentions to be vaccinated over a longer period of time (the so-called COSMO Germany study; Betsch et al., 2020). Betsch et al. (2021) results show that from April 2020–during which the intention to be vaccinated was around 79%–there was a steady decrease over the year 2020. The survey reports the lowest levels in early and mid-December, with only about 48% of the population reporting agreement toward COVID-19 vaccination. After this drop, support rose again to 68% by the beginning of March 2021. However, vaccination intentions were considerably lower in young adults (under 30s), and, perhaps even more worryingly, seem to be plateauing at this lower level since February^1^ (Betsch et al., 2021). In this context, it should be noted that vaccination intentions may rapidly change due to the emotional effects of popular media reports. Nevertheless, given the importance of young adults becoming vaccinated for reducing the number of infections, the present study examines whether their vaccination intentions are related to individual epistemic beliefs and to prosocial values, and also investigates possible mediator effects of vaccination safety and effectiveness beliefs.

### Epistemic Beliefs and Vaccination Intentions

Epistemic beliefs are individual, subjective views, conceptions and theories about the creation, ontology, meaning, justification and validity of scientific knowledge (Priemer, 2006). According to the framework by Hofer and Pintrich (1997), there are four dimensions of epistemic beliefs: *certainty of knowledge* (Does one perceive knowledge as either certain or either tentative?)*, simplicity of knowledge* (Does one perceive knowledge as either simple or either complex?)*, source of knowledge* (To what extent does one perceive knowledge to originate from the self respectively from external authorities?), and *justification for knowing* (How is knowledge justified?). Bråten et al. (2013) further specified the justification for knowing dimension by splitting it into three sub-dimensions: *justification by authority, personal justification*, and *justification by multiple sources*. Individuals high in justification by authority refer to authorities and their expertise to justify knowledge claims. Personal justification is about justifying knowledge claims based on one's personal opinions or feelings. In contrast to personal justification, justification by multiple sources implies an evaluation of knowledge claims by means of integrating and evaluating multiple sources (Greene et al., 2008). In this regard, Beck et al. (2020) found significant relationships between all three dimensions of justification for knowing and individual beliefs in COVID-19 related conspiracy theories. For example, in their study with 215 participants, justification by authority negatively correlated with beliefs in COVID-19 related conspiracy theories, whereas the corresponding relationship was positive for personal justification. Hence, knowing that justification for knowing is associated with individual opinions toward COVID-19 related topics (Beck et al., 2020), we concentrate on this dimension as a central predictor of individual vaccination intentions.

Not surprisingly, knowledge structures vary across domains. Therefore, epistemic beliefs are often conceptualized with regard to specific disciplines or domains (e.g., biology-specific epistemic beliefs; Muis et al., 2006; Rosman et al., 2020). It is believed, according to the Theory of Integrated Domains in Personal Epistemology (TIDE), that global epistemic beliefs influence academic beliefs, which again influence beliefs about specific domains or even topics (Merk et al., 2018). This influence goes both ways, also back from more specific to more global beliefs. The domain-specificity of epistemic beliefs thereby is challenging since researchers have to choose a specific level of investigation prior to conducting their study or building their theory. In this regard, Bråten and Strømsø (2010) argue that “personal epistemology at different levels of specificity may have strongest impact on facets of academic learning at comparable levels of specificity” (p. 640). As knowledge about SARS-CoV-2 mainly stems from the medical domain and since we were interested in an outcome related to this same domain (i.e., vaccination intentions), we focused, for the present study, on medicine-specific epistemic beliefs.

Epistemic beliefs strongly impact medical decision making, for example through their influence on which experts individuals choose to trust (Kienhues and Bromme, 2012). Furthermore, previous studies found negative relationships between scientific reasoning and anti-vaccination attitudes regarding vaccinations in general as well as vaccinations against COVID-19 (Cavojová et al., 2020). More specifically, individuals with better scientific reasoning abilities, who, for example, form their opinion based on reliable scientific information, had a more positive attitude toward vaccinations (Cavojová et al., 2020). Reliable scientific information on COVID-19 and COVID-19 vaccinations, in turn, is mostly given by medical experts (e.g., virologists, epidemiologists, or public health scholars), who represent an epistemic authority to laypeople in this area of expertise (Lavazza and Farina, 2020). Hence, if individuals believe that expertise and authority are important aspects of the knowledge generation process, they will more likely form their opinions regarding COVID-19 vaccinations based on reliable scientific information, which (to date [March 2021] as well as by the time the study was conducted [January–February 2021]), strongly suggest that the vaccinations are safe and effective. Turning to such information may therefore increase vaccination intentions. Hence, the present study hypothesizes that:

*H1:* There is a positive correlation between justification by authority and COVID-19 vaccination intentions.

### Prosocial Values and Vaccination Intentions

Because younger people are not threatened by SARS-CoV-2 to the same amount as the elderly, becoming vaccinated against the virus can be seen as an act of “voluntary behavior, meant to benefit another” (Padilla-Walker and Carlo, 2014, p. 6)–in short, a prosocial act. Prosocial behavior is thereby influenced by genetics, neurophysiological determinants, socialization, culture, and contextual factors. Furthermore, it is strongly associated with feelings of empathy and occurs more often with regard to close people (Padilla-Walker and Carlo, 2014). Personality traits such as agreeableness or the HEXACO variable honesty-humility (Hilbig et al., 2014) are known to be associated with prosocial behavior. A different approach to predicting prosocial behavior are human values. Values are the social representation of deeply rooted basic motivations, and therefore affect individual opinions, attitudes, and behavior. Sharing each other's values elicits a sense of connectedness between people (Wolf et al., 2020), which should lead to more prosocial behavior toward one another.

Schwartz (2003) defines 10 basic values (power, achievement, hedonism, stimulation, self-direction, universalism, benevolence, tradition, conformity, and security). Thereby, benevolence is the value associated with prosocial behavior, because it is about “preservation and enhancement of the welfare of people with whom one is in frequent personal contact” (Schwartz, 2003, p. 269). It describes helpful, honest, forgiving, loyal, and responsible behavior. Hence, people with strong benevolence values are more likely to act in prosocial ways than others.

As outlined above, becoming vaccinated is also a prosocial act since it not only protects oneself, but also one's social environment. This is especially true for younger people who have less risk of developing severe disease. Since prosocial values and prosocial behavior are closely associated (e.g., Wolf et al., 2020), the conclusion that prosocial values influence vaccination intentions is warranted. Empirically, this reasoning is supported by evidence on the connection between prosociality and the willingness to self-isolate in order to protect others in the context of the pandemic. For example, Wolf et al. (2020) identified self-transcendence values such as benevolence as an important factor in promoting prosocial pandemic-related behavior (e.g., social distancing). In addition, it seems that prosocial personality traits are associated with a greater compliance behavior (Heffner et al., 2021), and data analyses by Ghosh and Martcheva (2020) suggested that “prosocial awareness has competitive potential to flatten the curve” (p. 1). Furthermore, a study about polio vaccination in Israel showed that vaccination intentions directly depend on prosociality (Wells et al., 2020). In sum, these studies suggest that prosocial values have a huge impact on the willingness to do something to protect fellow humans. Based on these deliberations, the present study hypothesizes:

*H2*: There is a positive correlation between prosocial values and vaccination intentions.

### The Moderating Effects of Epistemic Beliefs on the Relationship Between Prosocial Values and Vaccination Intentions

Until now, we have discussed the separate potential effects of epistemic beliefs and prosocial values on vaccination intentions. However, one may also expect that both these variables interactively influence the will to become vaccinated. In fact, for prosocial values to positively affect vaccination intentions, it is important that individuals with such traits recognize that becoming vaccinated contributes to herd immunity and hence protects fellow humans. Evidence for this assumption comes from an online experiment by Betsch et al. (2013), who showed that an experimental group receiving information on herd immunity and social benefit through vaccinations were more likely to become vaccinated compared to a group not receiving such information. A more recent study by Betsch and Böhm (2018) confirmed these findings.

As outlined above, knowledge on the effects of vaccinations frequently stems from medical experts (e.g., virologists and epidemiologists). Hence, if individuals recognize such experts' authority regarding the knowledge generation process in medicine, they will more likely come to the conclusion that becoming vaccinated also protects fellow humans (e.g., Betsch et al., 2013). Strong beliefs in justification by authority may thus further strengthen the expected positive relationships between prosocial values and vaccination intentions. In contrast, if one does not know (or believe) that vaccinations not only protect oneself, but also others, prosocial values likely will not have much impact on vaccination intentions. Technically speaking, this reasoning is consistent with a moderator effect–hence we expect that epistemic beliefs moderate the relationship between prosocial values and vaccination intentions. We suggest the following hypothesis:

*H3*: There is an interaction between beliefs in justification by authority and prosocial values in their influence on vaccination intentions. The positive correlation between prosocial values and vaccination intentions becomes stronger with increasing beliefs in justification by authority.

### Additional Exploratory Analyses

In addition to testing the three aforementioned confirmatory hypotheses, we conducted a number of exploratory analyses. Among others, we tested whether the other two dimensions of justification beliefs (personal justification and justification by multiple sources) also relate to vaccination intentions. Thereby, we expected that personal justification, which is about rejecting authority and finding things out by oneself, is associated with lower vaccination intentions, and that the contrary would be true for justification by multiple sources, which describes an evidence-based approach to knowledge. Furthermore, we analyzed whether vaccination safety and effectiveness beliefs would mediate the relationships between epistemic beliefs and vaccination intentions. Such a mediator effect would be highly consistent with our theorizing on the effects of epistemic beliefs. In fact, as outlined above, we had expected that individuals with strong beliefs in justification by authority would more likely refer to reliable scientific information when deciding whether to get vaccinated–information that strongly speaks for the vaccinations being safe and effective. It should be noted that notwithstanding their consistency with our theory, we had not specified any of these expectations prior to collecting our data, which is why all corresponding analyses are exploratory.

## Method

### Participants and Procedure

#### Data Collection Procedures

Data were collected in a correlational cross-sectional online study. Hence, participants were not randomly assigned to a treatment, and there was no differentiation between a control and an experimental group. The online questionnaire was administered in German language and realized by means of the survey software EFS Survey (Unipark). Participants were recruited through a university mailing list and through social media groups (e.g., Facebook). They did not get any reward for their participation. While completing the questionnaire, participants were not aware of the research question or the study hypotheses. All study procedures were in full accordance with the declaration of Helsinki and the APA ethics code (American Psychological Association, 2002). At the beginning of the questionnaire, an informed consent page included information about the study's inclusion and exclusion criteria (see below) and indicated that participation was anonymous, voluntary, and that it may be terminated at any time. Explicit agreement to the terms specified on this page was mandatory for study participation.

#### Inclusion and Exclusion Criteria

The sample consisted of students from universities throughout Germany, regardless of their study discipline, age, gender or nationality. As outlined above, we opted for a student sample since young adults may, due to their increased mobility, more strongly contribute to herd immunity once they are vaccinated. Students who were either pregnant, had already been vaccinated against COVID-19, or had already had COVID-19 (as indicated by a positive test), were not eligible for participation as these factors may bias results due to their influence on vaccination intentions. In addition to informing participants about the inclusion and exclusion criteria on the informed consent page, the fulfillment of these criteria was verified one-by-one by means of a series of yes/no questions that were presented on a separate page. Furthermore, we aimed to exclude participants with major protocol deviations such as an implausibly fast questionnaire completion.

#### Sample Size Rationale

According to current literature, the lowest acceptable sample size for a multiple regression in a non-experimental design is 300 participants (Bujang et al., 2017). To be on the safe side with regard to our exclusion criteria, we aimed to recruit at least *N* = 350 participants.

#### Sample Description

Data collection started on January 22nd, 2021, and was terminated on February 1st, 2021. A total of *N* = 364 students agreed to participate in the survey (as indicated by the acceptance of the terms specified in the informed consent). In line with our inclusion and exclusion criteria, we excluded *n* = 50 participants who were either not enrolled at a university (*n* = 24), pregnant (*n* = 3), SARS-CoV-2 PCR test positive (*n* = 5), already vaccinated (*n* = 16), or had completed the questionnaire in <120 s (*n* = 2). The finale sample thus consisted of *N* = 314 participants aged 18–41 years (*M* = 26.10; *SD* = 55.61; 72.6% female, 27.1% male, 0.3% diverse).

### Variables

#### Epistemic Beliefs

To measure participants' epistemic beliefs, we focused, as outlined above, on justification for knowing (i.e., justification beliefs). In line with the framework by Bråten et al. (2013), we used a scale targeting justification by authority, personal justification, and justification by multiple sources (even though it should be noted that our confirmatory analyses focus on justification by authority alone). We thereby adapted the German version of the corresponding scale by Klopp and Stark (2016), originally developed in Norwegian language by Bråten et al. (2013). This questionnaire assesses justification beliefs on a 6-point Likert scale ranging from “do not agree at all” to “fully agree.” As outlined above, we measured epistemic beliefs regarding medicine for its content-related proximity to vaccination intentions. To do so, the items by Klopp and Stark (2016) were slightly adapted (e.g., the item “When I read something that is based on scientific investigations, then I know that it is correct” was changed to “When I read something that is based on medical science, then I know that it is correct”; English translation by the authors).

#### Prosocial Values

To measure prosocial values, we focused on Schwartz' (2003) construct of benevolence and the contrasting construct of hedonism (again, the latter was included for exploratory analyses only). Therefore, we used the corresponding subscales of the German version of the *Schwartz Portraits Value Questionnaire* (Schmidt et al., 2007). In this questionnaire, respondents are asked to rate their similarity to a hypothetical person on a 6-point scale ranging from “very dissimilar” to “very similar.”

#### Vaccination Intentions

Our outcome variable were vaccination intentions regarding COVID-19. We measured this by a single item asking participants how likely they would become vaccinated against COVID-19 when they had the possibility (“How would you decide when you had the possibility to be vaccinated against COVID-19 next week (given that enough vaccine doses are available for everyone)?”). Responses were given on a 7-point scale from 1 (“definitely not become vaccinated”) to 7 (“definitely become vaccinated”; English translations by the authors). We opted for a single item measurement since this item format seems to be the gold standard to date, and has already been used in multiple corresponding studies (e.g., Betsch et al., 2020, 2021; Faasse and Newby, 2020; Taylor et al., 2020; Kwok et al., 2021). The item wording was exactly the same as in Betsch et al. (2021), except for the notion “given that enough vaccine doses are available for everyone” in parentheses. We added this notion since we wanted to avoid that students, who usually have a lower probability of severe disease, negatively respond to the item because they would want their dose to be administered to at-risk groups (as there was a vaccine shortage in Germany by the time of the study).

#### Covariates

In addition to the main study constructs, we assessed perceived vaccination safety and effectiveness, knowledge on COVID-19 vaccines, the expected severity of an infection with COVID-19, and fear of COVID-19. These variables were included because of their potential influence on vaccination intentions, thus allowing for additional exploratory analyses (e.g., mediator analyses and partial correlations).

*Perceived vaccination safety* was measured by asking participants whether they believed that the currently approved vaccines were safe (“The currently approved vaccines (BioNTech, Moderna) are safe and do not have severe adverse effects”; 7-point scale ranging from 1 “do not agree at all” to 7 “fully agree”).

*Perceived vaccination effectiveness* was measured by two items. First, we asked participants whether they believed that vaccinated people are protected against SARS-CoV-2 (“Vaccinated people are well-protected against SARS-CoV-2”). Second, we asked whether they believe that the more people are vaccinated, non-vaccinated people will also be protected (“The more people are vaccinated against SARS-CoV-2, the more unvaccinated people will also be protected”). It is of note that by the time the study was conducted, there was not much empirical evidence on this “herd immunity” assumption, even though virologists and epidemiologists were generally optimistic in this regard. Both items' response formats were identical to the one of the single item on vaccination safety.

To measure *knowledge about COVID-19 vaccines*, we asked the participants what kind of vaccines the vaccines from BioNTech, Moderna, and Oxford/AstraZeneca are (response options: “inactivated vaccine,” “attenuated vaccine,” “gene-based vaccine (mRNA),” “vector-based vaccine,” “don't know”). Correct answers were scored with a 1, incorrect answers with a 0. Subsequently, scores over the three items were averaged, resulting in an indicator ranging from 0 (3 wrong answers) to 1 (3 correct answers).

To measure the *expected severity of an infection with COVID-19*, we asked participants how an infection would be for them–again on a seven-point scale from “harmless” to “dangerous.”

As a final exploratory measure, fear of COVID-19 was assessed using the 7-item *Fear of Coronavirus-19 Scale* (Ahorsu et al., 2020), which we translated to German (from English) for the present study. Response format was a 5-point scale ranging from “strongly disagree” to “strongly agree.”

All items were administered in German language. Furthermore, all items belonging to one questionnaire were presented in random order.

### Statistical Analyses

Hypotheses H1 and H2 were tested using Spearman correlation analysis. H3 was tested by means of a regression-based interaction analysis (Aiken and West, 1991). This was realized using the SPSS macro PROCESS (Hayes, 2018; model 1; independent variable: benevolence; moderator; justification by authority; dependent variable: vaccination intentions). For all analyses, inference criteria were *p* < 0.05.

## Results

A descriptive overview of the study variables can be found in Table 1. Since epistemic belief inventories often exhibit psychometric problems (DeBacker et al., 2008; Mason, 2016), we first conducted a confirmatory factor analysis (CFA) to test the dimensionality of our justification inventory. We thereby tested the three-factor model (justification by authority, personal justification, justification by multiple sources) against a one-factor baseline model. Results suggested a better fit of the three-factor model compared to the baseline model (CFI = 0.979; TLI = 0.968), and a good fit of the three-factor model overall ($\chi_{\text{df }=\;24}^{2}$ = 40.921, *p* = 0.017; RMSEA = 0.047; SRMR = 0.045). This confirms the expected three-factor structure of the inventory. Reliabilities of all scales employed in the study were good to acceptable, with the exception of the benevolence scale (α = 0.606), which was on the lower bound of what is generally considered acceptable (see Table 1).

**Table 1:** Descriptive statistics and intercorrelations of the main study variables.

	***M***	***SD***	**1**	**2**	**3**	**4**	**5**	**6**	**7**	**8**	**9**	**10**	**11**
1 Vaccination intention	5.175	2.140	-	0.387^**^	−0.458^**^	−0.230^**^	−0.002	−0.038	0.785^**^	0.691^**^	0.510^**^	0.094	0.135*
2 Justification by authority	4.483	0.796	0.339^**^	(0.762)	−0.349^**^	−0.314^**^	0.003	0.041	0.433^**^	0.438^**^	0.358^**^	−0.023	0.033
3 Personal justificaiton	2.321	0.958	−0.451^**^	−0.385^**^	(0.771)	0.235^**^	−0.046	0.110	−0.523^**^	−0.463^**^	−0.322^**^	0.002	−0.234^**^
4 Justification by multiple sources	4.801	0.896	−0.232^**^	−0.344^**^	0.188^**^	(0.728)	−0.066	0.121*	−0.314^**^	−0.259^**^	−0.261^**^	0.089	0.063
5 Benevolence	4.920	0.686	0.036	−0.002	−0.060	−0.054	(0.606)	0.210^**^	−0.050	0.029	0.095	0.086	0.012
6 Hedonism	4.510	0.914	−0.034	0.000	0.113*	0.141*	0.146^**^	(0.782)	−0.114*	−0.044	−0.030	0.020	−0.084
7 Perceived vaccination safety	4.854	1.730	0.752^**^	0.417^**^	−0.530^**^	−0.304^**^	−0.016	−0.121*	–	0.789^**^	0.580^**^	0.052	0.195^**^
8 Perceived vaccination effectiveness: Protects oneself	5.051	1.512	0.624^**^	0.400^**^	−0.452^**^	−0.254^**^	0.079	−0.033	0.721^**^	-	0.649^**^	0.012	0.147^**^
9 Perceived vaccination effectiveness: Protects others	4.707	1.752	0.442^**^	0.334^**^	−0.293^**^	−0.270^**^	0.103	−0.039	0.515^**^	0.575^**^	-	−0.012	0.012
10 Fear of Coronavirus-19 Scale	2.056	0.695	0.024	−0.065	0.019	0.068	0.098	−0.015	0.001	−0.062	−0.052	(0.817)	0.010
11 Prior knowledge about COVID-19 vaccines	0.466	0.348	0.168^**^	0.003	−0.226^**^	0.061	0.034	−0.098	0.220^**^	0.169^**^	0.002	0.003	(0.658)

N = 314; M, mean; SD, standard deviation; values in parentheses on the diagonal = Cronbach's Alpha; values above the diagonal = Pearson correlations; values below the diagonal = Spearman correlations; *p < 0.05 (two-tailed);

** *p < 0.01 (two-tailed)*.

### Confirmatory Hypothesis Tests

Hypothesis 1 posits a positive relationship between justification by authority and vaccination intentions. In line with this expectation, we found a significant Spearman correlation between the two variables (*r* = 0.339; *p* < 0.001). According to common rules of thumb, this indicates a moderate effect size. Hypothesis 1 is confirmed.

With regard to Hypothesis 2, we expected a positive relationship between prosocial values (i.e., benevolence) and vaccination intentions. Contrary to our expectations, we found no significant correlation between benevolence and vaccination intentions (*r* = 0.036; *p* = 0.525). Therefore, Hypothesis 2 is not confirmed. Considering this non-significant result, we additionally conduced a sensitivity analysis to investigate possible issues of statistical power. Using G^*^Power 3.1 (Faul et al., 2009), we thereby found that a sample size of *N* = 314 is sufficient to detect an effect of ρ = 0.184 with a probability (i.e., 1–β) of 0.95, or an effect of ρ = 0.140 with a probability of 0.80. Effects above ρ = 0.20 would have been detected with a very high probability (1–β > 0.97). Considering that effect sizes under ρ = 0.20 have little practical meaning, we conclude that our analyses regarding Hypothesis 2 were not underpowered.

Hypothesis 3 suggests that there is an interaction between justification by authority and benevolence in their influence on vaccination intentions. We thereby expected that the (positive) correlation between benevolence and vaccination intentions would increase with rising beliefs in justification by authority. Contrary to our expectations, no corresponding interaction was found–the increase in *R*^2^ after adding the product term of benevolence and justification by authority to the regression equation was very low (Δ*R*^2^ = 0.001) and not significant [*F*_(1, 310)_ = 0.430, *p* = 0.513]. Hypothesis 3 is not confirmed.

To ensure the robustness of these results, we additionally retested all three hypotheses using binary logistic regression (DV coding: scale values 1–3 = 0 and 5–7 = 1, middle category omitted). Furthermore, we retested Hypotheses 1 and 2 using ordinal logistic regression. The pattern of results (i.e., Hypothesis 1 confirmed, Hypotheses 2 and 3 not confirmed) thereby was identical across all analyses.

### Exploratory Analyses

We followed up with an analysis of our exploratory research questions. First, we tested whether the positive relationship between justification by authority and vaccination intentions might be confounded by third variables. To do so, we conducted a Spearman partial correlation between justification by authority and vaccination intentions controlling for age, gender, prior knowledge about COVID-19 vaccines, and fear of COVID-19 (see section Covariates). Results showed that the correlation remained significant when controlling for the aforementioned variables (*r* = 0.356; *p* < 0.001), thus indicating that the relationship between justification by authority and vaccination intentions is not confounded by these variables.

Second, in line with our expectations on the effects of the other two justification scales (see above), we found a significant negative correlation between personal justification and vaccination intentions (*r* = −0.451; *p* < 0.001), indicating a moderate to high effect size. Moreover, contrary to what we would have expected, we found a significant, albeit rather low, negative correlation between justification by multiple sources and vaccination intentions (*r* = −0.232; *p* < 0.01). With regard to human values, we found no significant relationship between hedonism and vaccination intentions–based on our theorizing regarding Hypothesis 2, we would have expected a negative correlation. Finally, we found a small but significant positive relationship between knowledge on COVID-19 vaccines and vaccination intentions (*r* = 0.168; *p* < 0.01).

In addition, as this was highly consistent with our theorizing (see above), we conducted a mediator analysis to investigate whether beliefs in vaccine safety and effectiveness would mediate the relationship between justification by authority and vaccination intentions. This analysis was conducted by setting up a model with three parallel mediators in the SPSS macro PROCESS (Hayes, 2018; model 4; independent variable: justification by authority; mediators: perceived vaccination safety, perceived vaccination effectiveness in protecting oneself, perceived vaccination effectiveness regarding herd immunity; dependent variable: vaccination intentions). This analysis revealed highly significant indirect effects of perceived vaccination safety (*B* = 0.726; 95% bootstrap CI [0.527; 0.955]) and perceived vaccination effectiveness in protecting oneself (*B* = 0.193; 95% bootstrap CI [0.018; 0.391]), whereas no significant effects were observed with regard to perceived vaccination effectiveness to protect others (*B* = 0.029; 95% bootstrap CI [−0.068; 0.126]). After the inclusion of these mediator variables in the model, the direct effect of justification by authority on vaccination intentions became non-significant (*B* = 0.092; *p* = 0.382), thus indicating full mediation. This assumption of full mediation was corroborated by significant Sobel tests (perceived vaccination safety: *z* = 6.664; *p* < 0.001; perceived vaccination effectiveness in protecting oneself: *z* = 2.530; *p* < 0.05). Hence, we conclude that perceptions of vaccination safety and effectiveness in protecting oneself fully mediate the relationship between justification by authority and vaccination intentions.

## Discussion

This study aimed to investigate the relationship between epistemic beliefs, prosocial human values, and vaccination intentions at the start of the COVID-19 vaccination campaign in Germany. We thereby focused on university students since they could play an important role in attaining herd immunity due to their increased mobility and sociability. Data were collected in a cross-sectional correlational online study, using established measures on epistemic beliefs, human values, and vaccination intentions.

### Main Findings

Confirming our first hypothesis, we found that individuals who believe in expertise and authority as important aspects of the knowledge generation process (in medicine) report increased vaccination intentions. This may be because medical experts (e.g., virologists, epidemiologists, or public health scholars), at least at the time of data collection, almost unanimously spoke in favor of the safety and effectiveness of the available COVID-19 vaccines. This finding is in line with prior research by Cavojová et al. (2020), who found that individuals had a more positive attitude toward vaccinations when forming their opinions based on reliable scientific information. Furthermore, it is in line with findings on the acceptance of COVID-19 vaccinations being strongly associated with trust in (biomedical) research (Palamenghi et al., 2020).

However, contrary to what we had expected in Hypothesis 2, the data revealed no significant correlation between human values and vaccination intentions. This is surprising as it contradicts the findings by Wells et al. (2020), who found evidence for a corresponding relationship. However, it should be noted that their study focused on polio vaccination. The polio vaccination campaign has been ongoing since the 1950s and the severe consequences of polio disease as well as the effects of corresponding vaccinations are well-known (Blume and Geesink, 2000). COVID-19, on the other hand, is a novel disease, with newly developed vaccines. Therefore, at least by the time of data collection, there was no scientific consensus on whether vaccinated individuals may still transmit the disease (Connors et al., 2021). In fact, at the beginning of 2021, the available data suggested that asymptomatic transmission of the virus could not be ruled out despite vaccination (e.g., Bleier et al., 2021; Connors et al., 2021). Considering that acknowledging the benefit of vaccinations regarding the protection of one's social environment is a necessary condition for prosocial values to have an effect on vaccination intentions, this could thus well explain why we found no correlation between prosocial values and vaccination intentions. Such an explanation is in line with the findings by Betsch et al. (2013), which suggest that knowledge about a potential herd immunity determines the relationship between prosocial values and vaccination intentions. What speaks against this interpretation is that vaccinations reduce the probability of suffering from severe COVID-19 (e.g., Bleier et al., 2021; Connors et al., 2021), thus lowering the burden on the health care system, a circumstance from which others may well benefit. However, as prosocial values primarily impact one's behavior toward “people with whom one is in frequent personal contact” (Schwartz, 2003, p. 269), this rather indirect effect may not have been perceived as “prosocial” compared to a direct protection of one's social environment. In this regard, testing the effects of more general worldviews, as suggested by cultural theory (e.g., individualism or egalitarianism; Douglas, 1966; Michaud et al., 2009) might be a fruitful endeavor for future research. Finally, another possible explanation for not finding a relationship between prosocial values and vaccination intentions can be derived from the wording of our item on vaccination intentions. In fact, respondents answered based on the assumption that vaccination was available to everyone. Hence, prosociality may not have been stimulated since our participants might have expected that in this hypothetical scenario, at-risk individuals would have the possibility of protecting themselves, which would also reduce the “prosocial” benefits of younger people becoming vaccinated.

With regard to Hypothesis 3, we found no significant moderator effect of justification by authority on the relationship between benevolence and vaccination intentions. Since multiple regression analyses with interaction terms require rather large samples and since *N* = 300 is usually considered the lower bound of what is acceptable, power issues may have played a role in this non-significant result. However, it should also be noted that without a significant bivariate relationship between benevolence and vaccination intentions (see Hypothesis 2), an interaction between benevolence and justification by authority becomes unlikely for theoretical reasons. In fact, the same reasons for not finding a positive relationship between benevolence and vaccination intentions might have led to us not finding evidence for a moderator effect of justification by authority on the relationship between prosocial values and vaccination intentions. Again, the lack of a scientific consensus (by the time of data collection) on the protection of others through vaccination may have led to even those individuals who value expertise and authority to not recognize the “prosocial” benefits of vaccinations. This absence of a moderator effect thus strengthens our argumentation in the last paragraph–even though it should be taken into account that interpreting non-significant findings is inherently difficult for statistical reasons.

With regard to our exploratory analyses, the negative correlations between personal justification respectively justification by multiple sources and vaccination intentions warrant some further attention. Individuals with strong beliefs in personal justification value a knowledge generation process based on their personal views and opinions (Bråten et al., 2013), which implies a rejection of the scientific method as a whole. Hence, they might have succumbed to a rather abstract feeling of doubt regarding the safety and effectiveness of the “new” vaccines, not acknowledging the rather favorable scientific evidence. With regard to justification by multiple sources, we were somewhat surprised by the negative correlations with vaccination intentions. This was because considering and evaluating multiple sources of evidence is usually seen as a nuanced and desirable approach to information (e.g., Bråten et al., 2013). However, in this specific case, high beliefs in justification by multiple sources might have led to individuals rejecting the (almost unanimously positive) “mainstream” information on COVID-19 vaccinations by referring, for example, to anti-vaccination sites or dubious social media channels. Furthermore, high justification by multiple sources might have impaired trust in COVID-19 related science since individuals who consult a multitude of sources more likely become aware of scientific disagreements on the response to the pandemic (e.g., Farina and Lavazza, 2020). This, in turn, might have led to reduced vaccine safety and effectiveness beliefs, thus lowering vaccination intentions. Interestingly, such arguments are in line with the findings by Beck et al. (2020), who found that justification by multiple sources positively correlates with beliefs in COVID-19 related conspiracy theories. However, since we did not measure the types of sources that our participants referred to, future research on these relationships is required.

In an additional exploratory analysis, we followed up on the potential mechanisms behind the relationship between justification by authority and vaccination intentions. We thereby found that perceptions of vaccination safety and effectiveness (in protecting the vaccinee) fully mediate the relationship between justification by authority and vaccination intentions. To our knowledge, this is the first study providing evidence for a corresponding mediation. Though this finding is exploratory and has to be tested in (preferably experimental) follow-up studies, it is particularly important since it establishes a direct link between beliefs about the nature of medical knowledge and vaccination intentions through its influence on vaccine-related safety and effectiveness beliefs–thus underlining how important trust in authorities is in influencing behavioral intentions. In addition, this mediator effect further substantiates our theoretical assumptions on the effects of justification by authority and thus increases the robustness of our evidence. Connecting these findings with our exploratory results on the effects of justification by multiple sources, future research may consider different source types that individuals refer to as another (serial) mediator which predicts vaccination safety and effectiveness beliefs. Such a model would provide additional insights on what determines vaccination intentions through vaccination safety and effectiveness beliefs, which we see, because of its enormously important practical implications, as a promising avenue for future research.

### Strengths and Limitations

First, it is important to note that our study employed a correlational design, which allows no causal inferences. For example, the positive relationship between vaccination intentions and justification by authority might be caused by an unknown third variable. However, it should also be noted that our findings are consistent with the literature, and that our mediator analysis perfectly fits our theoretical assumptions. Notwithstanding this, future research, preferably using experimental and/or longitudinal designs, is warranted.

Second, the generalizability of our findings is limited by the possible influence of social desirability. Furthermore, psychology has long established that intention and behavior are two distinct concepts and that intentions may not always lead to corresponding behaviors (e.g., Ajzen, 1991; Ajzen and Schmidt, 2020). Of course, we were not able to assess whether participants who affirmed their intention to be vaccinated would actually get themselves vaccinated. It should also be noted that, with some rare exceptions, the scenario of young adults becoming vaccinated was hypothetical at the time of data collection due to vaccine shortages. In addition, vaccination intentions, vaccine safety and effectiveness beliefs, as well as trust in science are subject to a rather strong variability (e.g., due to changes in media coverage), which is why justification by authority is likely just one factor among many to influence vaccination intentions. For these reasons, caution is warranted when interpreting our findings.

Furthermore, it should be noted that our sample consisted of a rather small number of university students, and that our findings might differ with regard to other relatively young age groups (e.g., apprentices). In addition, and while we think that a vaccination of the student-age population is absolutely crucial in the upcoming stage of the vaccination campaigns in Europe, investigating other age groups such as the elderly is important, too. Tentatively, we would argue that justification by authority might have even stronger effects in the elderly. In fact, high justification by authority would lead them to quickly realize the extremely favorable risk-benefit ratio of all COVID-19 vaccines in their age groups, hence likely inducing even stronger vaccination intentions compared to younger people. In order to be able to draw conclusions on a larger scale, further research, with larger sample sizes, a more heterogeneous (and preferably international) set of participants, and different recruiting modes, is necessary.

### Implications

A major strength of our study is the consistency of our results to the theoretical assumptions on the potential effects of epistemic beliefs on vaccination intentions. Using a mediator analysis, we showed that justification by authority influences beliefs in the safety and effectiveness of COVID-19 vaccines, which, in turn, influences vaccination intentions. We derive two main implications from these findings. First, public perceptions of expertise and authority are extremely important with regard to the vaccination campaign. If individuals acknowledge the crucial role of scientists and public health experts in justifying COVID-19 related knowledge claims, they will, through increased safety and effectiveness beliefs, be more willing to become vaccinated against the disease. For this reason, science and public health communication should be a key element of each and every country's COVID-19 response strategy (see also Rosman et al., 2021). Openness and transparency have long been suggested as a central factor in building trust, which is why we would advocate for an honest, integer and transparent communication strategy. A second implication concerns the communication of potential side-effects of the vaccines. If authorities question the safety of a vaccine (either by direct communication or indirectly through limiting its use), this has considerable potential to reduce the vaccination willingness of the population–particularly in those who value expertise and authority. In this regard, it is of note that the safety of the AstraZeneca vaccine was called into question by mid-March 2021, with several countries temporarily suspending its use. At the same time, politicians and public health experts were quick to reassure the public that all COVID-19 vaccines are safe and effective. We know from the early phases of the pandemic that such conflicting messages are particularly challenging for the public (e.g., Goldstein et al., 2020). They also bear the risk that the population increasingly loses faith in governmental institutions, a trend that has been accelerating in Germany since the beginning of 2021 (Betsch et al., 2021). This brings us back to the beginning of this paragraph: If the public no longer believes in expertise as a justification for the response to the pandemic, controlling COVID-19 becomes impossible–be it through vaccinations, testing, masks, or non-pharmaceutical interventions. Therefore, effective crisis communication is now more important than ever.

## Data Availability Statement

The raw data supporting the conclusions of this article will be made available by the authors, without undue reservation.

## Ethics Statement

Ethical review and approval was not required for the study on human participants in accordance with the local legislation and institutional requirements. The patients/participants provided their written informed consent to participate in this study.

## Author Contributions

This study was conducted in a research-oriented psychology course (Master track) at the Psychology Department of the University of Trier. The course was supervised by TR. Based on an initial idea developed during the course, TR conceived, planned, and supervised the study as well as the writing of the manuscript. The co-authors conducted the study, tested the confirmatory hypotheses, and wrote sections of an initial manuscript draft. TR substantially revised this draft and re-wrote several parts of the manuscript. Moreover, TR conducted a large part of the exploratory analyses and prepared the manuscript for publication. All authors have approved the final version of the manuscript to be published.

## Conflict of Interest

The authors declare that the research was conducted in the absence of any commercial or financial relationships that could be construed as a potential conflict of interest.

## Footnotes

^1^The present article was written in mid-March 2021, which is why this claim only applies to the time span between February and March.
